# Water Exercise and Quality of Life in Pregnancy: A Randomised Clinical Trial

**DOI:** 10.3390/ijerph17041288

**Published:** 2020-02-17

**Authors:** Raquel Rodríguez-Blanque, María José Aguilar-Cordero, Ana Eugenia Marín-Jiménez, María José Menor-Rodríguez, Maria Montiel-Troya, Juan Carlos Sánchez-García

**Affiliations:** 1 Research Group CTS1068, Andalusia Research Plan, Junta de Andalucía, San Cecilio Clinical Hospital, School of Nursing, Faculty of Health Sciences, University of Granada, 18071 Granada, Spain; 2 Research Group CTS 367, Andalusia Research Plan, Junta de Andalucía, School of Nursing, School of Health Sciences, University of Granada, San Cecilio Clinical Hospital, 18071 Granada, Spain; mariajaguilar@telefonica.net; 3 Research Group CTS1068, Andalusia Research Plan, Junta de Andalucía, Quantitative Methods for the Economics and Enterprise, Faculty of Economics and Business Sciences, University of Granada, 18071 Granada, Spain; anamarin@ugr.es; 4 Research Group CTS367, Andalusia Research Plan, Junta de Andalucía, Área Sanitaria de Ourense, 32616 Ourense, Spain; maria.jose.menor.rodriguez@sergas.es; 5 Research Group CTS1068, Andalusia Research Plan, Junta de Andalucía, School of Nursing Ceuta Campus, Faculty of Health Sciences, University of Granada, 51001 Ceuta, Spain; mariamontiel@ugr.es; 6 Research Group CTS1068, Andalusia Research Plan, Junta de Andalucía, School of Nursing, Faculty of Health Sciences, University of Granada, 18071 Granada, Spain; jsangar@ugr.es

**Keywords:** Pregnant woman, exercise, physical activity, quality of life

## Abstract

*Background*: Physical exercise helps to maintain a healthy lifestyle and its practice is recommended for women during pregnancy as a means of limiting the negative effects on the body that may take place and to optimise well-being, mood and sleep patterns, as well as encouraging daily physical activity, enhancing the ability to work and preventing pregnancy-related complications. *Aim*: To analyse the quality of life in pregnancy for women who complete a programme of moderate physical activity in water, following a designed method that the woman can perform physical exercise safely during pregnancy called the SWEP (study of water exercise during pregnancy) method. *Materials and methods*: A randomised clinical trial was performed. One hundred and twenty-nine pregnant women were randomly assigned either to an exercise class following the SWEP method (EG, *n* = 65) or to a control group (CG, *n* = 64). The trial began in week 20 of pregnancy (May 2016) and ended in week 37 (October 2016). Heath-related quality of life (HRQoL) was evaluated with the SF36v2 health questionnaire at weeks 12 and 35 of pregnancy. *Results*: The HRQoL score decreased significantly between weeks 12 and 35 of gestation, except for the mental health component, which in the CG fell by −3.28 points and in the EG increased slightly (*p* > 0.05). Among the CG, the score for the mental health component at week 35 was ≤42, indicating a positive screening risk of depression (39.20 ± 4.16). *Conclusions*: Physical activity programmes in water, such as SWEP, enhance the HRQoL of pregnant women.

## 1. Introduction

The World Health Organization has defined quality of life as “an individual’s perception of their position in life in the context of the culture and value systems in which they live and in relation to their goals, expectations, standards and concerns. It is a broad ranging concept affected in a complex way by the person’s physical health, psychological state, personal beliefs, social relationships and their relationship to salient features of their environment” [1].

Physical exercise has unquestionable health benefits, achieving risk reductions of at least 20%–30% for premature mortality and for over 25 chronic medical conditions [2].

Moreover, it enhances wellbeing by raising levels of beta-endorphins in the body. In pregnancy and childbirth, prior exercise can facilitate delivery and prevent complications [3,4]. Although the American College of Obstetrics and Gynaecology (ACOG) recommends that pregnant women should perform at least 30 minutes of moderate exercise, five days per week [5], it is observed that studies have shown that regular physical activity tends to decrease during pregnancy [6,7,8], despite its proven beneficial effects for the mother and for the foetus [9]. This change in behaviour may be caused by doubts among mothers and perhaps also among health care professionals about the advisability of physical exercise during pregnancy, the most appropriate type of exercise, and its frequency, intensity and duration [10,11,12] There are worries that physical activity might contribute to the mother suffering a miscarriage. Other justifications for becoming less physically active include the nausea experienced during pregnancy, together with increased body weight and size [13]. Moreover, the decrease in levels of physical activity during pregnancy tends to persist up for to six months after delivery [14].

Physical exercise helps maintain a healthy lifestyle and its practice is recommended for women during pregnancy as a means of limiting the negative effects on the body that may take place. Aquarobic exercise programmes for pregnant women have been shown to improve their preparation and physical development and to optimise well-being, mood and sleep patterns, as well as encouraging daily physical activity, enhancing the ability to work and preventing pregnancy-related complications [15]. The SWEP (study of water exercise during pregnancy) method is a programme of strength and endurance exercises, performed in water, that is specially designed for pregnant women [8] and intended to help them achieve the benefits described above.

The study hypothesis is that the practice of moderate physical exercise in water, following the SWEP guidelines, during weeks 20 to 37 of pregnancy, is associated with a better score in the SF36v2 health questionnaire and therefore enhances the health-related quality of life (HRQoL).

### Study Aim

To analyse the quality of life in pregnant women who complete a programme of moderate physical activity in water.

## 2. Materials and Methods

### 2.1. Study Design

In this randomised open-label clinical trial, the women and the researchers were all aware of the intervention. The trial was conducted in accordance with the CONSORT guidelines, published in 2010 [16] and approved by the Granada Research Ethics Committee (CEI-Granada), number 2606.2013.

Before the study, all the women taking part signed an informed consent form, in accordance with the Declaration of Helsinki, as revised by the Secretariat of the World Medical Association on 5 May 2015 (“WMA Declaration of Helsinki - Ethical Principles for Medical Research Involving Human Subjects,” 2013). The study is registered on the ClinicalTrials.gov website as number NCT02761967.

### 2.2. Participants

The women were recruited in the study in the first half of April of 2016, when they were in week 12 of gestation. This was done at the health clinics of the Granada-Metropolitan Health District (Granada, Spain) in order to correctly date the pregnancy. In an initial telephone contact with the research team, the women were informed about the study, and those who expressed interest in the project were sent an email with detailed information.

The researcher responsible for recruitment contacted 364 pregnant women, of whom 224 (61.54%) were subsequently excluded from consideration; 122 (33.52%) presented exclusion criteria (due to the fact that they presented some of the absolute contraindications for carrying out physical exercise during pregnancy, described in Box 1 of the ACOG document number 650 [5]), a further 82 (22.53%) refused to participate, mainly due to worries about harm from physical exercise during pregnancy and another 20 (5.49%) declined to take part for other reasons, including unavoidable family responsibilities or lack of time due to their employment.

**Figure 1:**
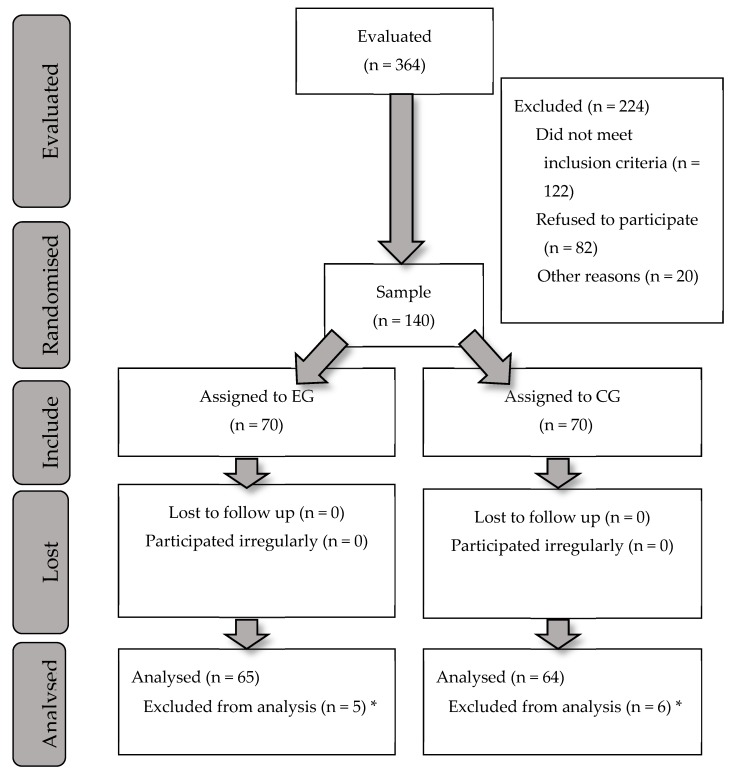
Flow diagram. * The delivery did not take place at the Granada Hospital Complex.

The study sample, thus, finally consisted of 140 women, aged between 21 and 43 years, who were randomly assigned either to the exercise group (EG) following the SWEP method (*n* = 70) or to the control group (CG) (*n* = 70). However, during the intervention, five women from the EG and six from the CG were lost to the study, and so the final sample was composed of 129 women, EG = 65 and CG = 64 (see Figure 1).

The following inclusion criteria were applied: healthy pregnant women, presenting none of the absolute contraindications described in Section 1 of ACOG document No. 650 (5): heart disease, restrictive lung disease, cerclage for cervical incompetence, multiple gestation with risk of preterm birth, second or third trimester persistent bleeding, preterm labour during current pregnancy, rupture of membranes, preeclampsia, pregnancy-induced hypertension and severe anaemia. If a relative contraindication was present, a favourable report was required from the women’s obstetrician to participate in the study.

If there were signs suggesting that exercise during pregnancy should be suspended, the women’s gynaecologist was consulted about the advisability of continuing with the exercise programme.

**Exclusion criteria.** Women who had taken regular physical exercise (planned and directed to improve their physical conditions) during the last 12 months or who had had a multiple pregnancy. Women who failed to attend at least 80% of the 54 sessions were also excluded from the study analysis.

### 2.3. Intervention

The EG carried out moderate physical activity in water, in the pool made available by the Faculty of Physical Activity and Sports Sciences of the University of Granada. Moderate exercise was understood to be between the score 12 and 14, represented as “somewhat hard”, according to the classical scale of Borg of perceived effort (EEP) [17]. The activity was supervised by midwives, nurses and graduates in sports science, who had previously received a training course in the SWEP method.

The exercise was designed specifically for this project, during weeks 20 to 37 of gestation. The program consisted of three sessions per week, each lasting 60 minutes. The sessions were composed of three phases: warm-up, main phase (with an aerobic element, followed by strength and endurance exercises) and final stretching and relaxation [18].

The women in both groups (EG and CG) received verbal and written dietary advice during pregnancy, including the following recommendations:

- Consume foods that are natural, varied, nutritious and light, in quantities to ensure appropriate weight gain, distributed among five or more light meals a day.
- Use salt in moderation (preferably iodised). The oil used should be extra virgin olive oil, and in dressings rather than fried.
- Eat 3–5 servings of fruit a day, plus one of vegetables and salad.
- Consume proteins 4–5 times a week, alternating legumes, eggs, meat and fish.
- Drink 1.5 to 2 litres of water a day.
- Wholemeal bread, pasta, rice and flour are preferable to processed varieties.
- Avoid or reduce the consumption of fried foods, animal fats, precooked meals, sweets and pastries.
- Avoid or reduce the consumption of soft drinks, tea, coffee and processed fruit juices.

Once the randomised groups were made, baseline data and the first questionnaire were collected from both groups. The EG started the studio and the CG received the standard recommendations during pregnancy, including guidelines from the midwife on the positive effects of physical exercise. These participants received the usual visits from healthcare providers (midwives, obstetricians and family doctor) during pregnancy, as did those in the EG. The same regular recommendations were given to both groups. The CG women were cited again to collect the final data.

### 2.4. Evaluation

#### 2.4.1. Sociodemographic and Anthropometric Variables

The following parameters were determined: age, level of studies, social class, previous children, marital status, obstetric formula, height, weight was measured with a Seca electronic balance (precission: 50 gr) in the first and third trimesters and parity, together with family and personal background.

Body weight (kg) was evaluated with a calibrated scale, at weeks 12 and 36 of pregnancy, because it was interesting to know the evolution of the BMI before the beginning of the intervention and the end of the intervention. Height (m) was measured using a calibrated metal rod.

#### 2.4.2. Perinatal Results

The following data were extracted from the partograph: reasons for admission, start mode, intact perineum after delivery, episiotomy, tears, gestation time (days), neonatal birthweight and the sex of the baby [19,20].

#### 2.4.3. Physical Activity Questionnaire

To assess the level of physical activity of the pregnant woman at the beginning of the study, in the 12th week of gestation, the Global Physical Activity Questionnaire (GPAQ) was used. The physical activity dimensions reported by the GPAQ were used, displacement and free time. The intensity level of physical activity was classified as moderate or vigorous in the dimensions of work and free time, and only in a moderate level for the displacement dimension. The questionnaire also included the report of the participant’s sedentary behaviour. The determination of the level of physical activity according to GPAQ was made according to the women’s report of "a typical week day"[21,22].

#### 2.4.4. Effort Exerted and Intensity of the Exercise

The effort exerted by the women during physical exercise was measured on the Borg rating of perceived exertion (RPE) scale [17]. The exercise programme was designed to produce a maximum score of 12–14 on this scale (“somewhat hard”), thus remaining within a moderate level of exertion, in accordance with ACOG recommendations [5].

Heart rates and levels of oxygen saturation during the training sessions were monitored using a Quirumed OXYM2000 portable pulse meter. The pulse was determined at the end of each exercise for women who reported a Borg RPE score >14.

#### 2.4.5. Health-Related Quality of Life

HRQoL was assessed by the short-form health questionnaire (SF36v2), a generic measure designed to assess an individual’s perceived health status [23,24]. The results of this questionnaire are scored from 0 (worst health or “poor”) to 100 (best or “excellent”), and address eight dimensions of health: physical functioning (PF), role participation with physical health problems (role-physical, RP), bodily pain (BP), general health (GH), vitality (VT), social role functioning (SF), emotional role functioning (RE) and mental health (MH).

Two overall results were obtained. The first is the summary score for the physical component (PCS), which at the lowest level indicates substantial limitations in self-care, physical, social and role activities, with severe bodily pain, frequent fatigue and general health classified as “poor”, while the highest level is indicative of an absence of physical limitations, disabilities and impaired well-being, with high levels of energy and overall health rated as “excellent”.

The second summary score is for the mental component (MCS). The lowest level for this concept indicates frequent psychological malaise, social incapacity and major role-playing deficiencies, due to emotional problems and to a state of mental health that in general is classified as “poor”. The highest level indicates frequent positive feelings, and an absence of psychological anguish and of limitations to normal social/role-playing activities arising from emotional problems. In this case, the individual’s general state of mental health is classed as “excellent”.

The questionnaire was distributed and completed in week 12 of pregnancy (baseline data) and again in week 35, i.e., in the first and third trimesters of pregnancy.

### 2.5. Sample Size

The sample size was calculated taking into account the findings of previous research in this area, based on a programme of physical exercise for pregnant women. The main variable considered was the prevalence of spontaneous birth in the study cohort. According to the results obtained by Price et al. [25], the prevalence of spontaneous birth should increase from 61.2% to 87% with the practice of physical exercise during pregnancy. To obtain a power of 80% to detect differences in the null hypothesis test H_0_: μ_1_ = μ_2_ by applying a chi-square test, for a 5% level of significance, and a joint standard deviation of 2.67, we calculated that 45 women per group, 90 in total, would need to be included in the study.

Study group assignment was random, probabilistic, without replacement and open label. The participants and the researchers were all fully aware of the different phases of the intervention. The identification data of each participant who met the inclusion criteria were noted on a ticket by one of the researchers. Each ticket was placed in an opaque envelope and each envelope in a container. Once all the envelopes were in the container, the principal investigator removed 81, which were assigned to the EG, and the remaining 81 were assigned to the CG.

### 2.6. Randomisation

Study group assignment was random. The participants and the researchers were all fully aware of the different phases of the intervention. The identification data of each woman recruited and who met the inclusion criteria was noted on a ticket by one of the researchers. Each ticket was placed in an opaque envelope and each envelope in a container. Once all the envelopes were in the container, the principal investigator removed 70, which were assigned to the EG, and the remaining 70 were assigned to the CG.

### 2.7. Statistical Analysis

The numerical variables are described by mean values and standard deviations, or by median values and percentiles in the nonparametric cases. For the qualitative variables, absolute and relative frequencies were calculated. The normality of the numerical variables was ascertained by the Kolmogorov–Smirnov test.

The differences between the exercise and control groups, with respect to the continuous variables, were examined by Student’s t-test, or by the Mann–Whitney U test when the distribution was non-normal. For qualitative variables, chi-square test or Fisher exact test was applied to determine differences between groups.

The changes over time, among and between the groups were examined by means of an ANOVA of repeated measures, taking time as the intrasubject factor and the group as the intersubject factor.

Quality Metric Incorporated software was used to obtain frequency.

All statistical analyses were performed using IBM SPSS 19 statistical software (SPSS Inc., Chicago, IL, USA). The significance level was set at *p* < 0.05.

## 3. Results

The participants joined the programme in week 20 of pregnancy (May 2016) and concluded it in week 37.

With respect to the baseline characteristics of the sample, there were no statistically significant differences between EG and CG in terms of body dimensions, history of previous births or areas of HRQoL (see Table 1).

Following previous studies, an analysis-by-protocol approach was taken to analyse the study data. The gestation time of each participant was determined by taking the value in weeks reflected in the corresponding partograph and converting the value to days of gestation.

Table 2 shows that there were no statistically significant differences between the EG and CG for gestation time (days) and neonatal birthweight. However, statistically significant differences were observed with respect to the status of the perineum, which remained intact for 26.15% of the women in the EG, versus 3.12% of those in the CG (*p* < 0.001).

Table 3 shows there were statistically significant differences over time and between the groups for most of the components, except for mental health.

Examination of the HRQoL summary scores shows that for the physical health component the EG score had decreased by 3.93 points by the end of the study period, whereas the CG, over the same period, had experienced a corresponding decrease of 8.07 points. For the mental health component, the CG score fell to below 42 points, which is classed as the first threshold in screening for depression (values ≤ 42 indicate risk of depression).

The analysis performed using the Quality Metric Incorporated software showed that the women in the CG presented a 73% risk of depression, compared to 44% for those in the EG.

Between the start of the study period and week 35 of pregnancy, all components of HRQoL worsened for both study groups, except mental health (in the CG, the latter score fell by >3 points, but the difference was not statistically significant).

**Figure 2:**
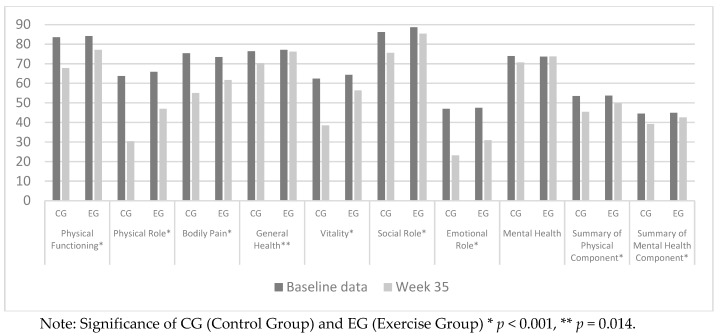
SF36v2 scores for CG vs. EG.

Figure 2 shows the changing patterns of scores obtained for the SF36v2 questionnaire, for EG and CG, from weeks 12 to 35 of pregnancy.

For both the mental and the physical summary components, and for all the individual components of the SF36v2 questionnaire except mental health, significant differences were observed between the two groups. The decrease in mean HRQoL scores between weeks 12 and 35 of pregnancy was significantly less among the EG participants than in the CG. Indeed, for the EG, only the components physical role, emotional role, physical functioning and mental health scored below 50 points at the end of the study period. With respect to general health, the women in both groups presented a fairly positive score, which improved in the EG and worsened only slightly in the CG. The differences between the two were not statistically significant.

The women in the CG experienced considerably sharper decreases in scores than those in the EG for the following HRQoL concepts: physical role (−33.3 vs. −18.94 points, respectively), bodily pain (−20.34 vs. −11.72 points), vitality (−23.92 vs. −7.98 points) and emotional role (−23.82 vs. −16.53 points).

## 4. Discussion

Gustafsson et al. [26] conducted a randomised controlled trial (RCT) of 855 healthy pregnant Caucasian women, with an EG that performed a 12-week exercise programme, between weeks 20 and 36 of pregnancy, in weekly group sessions led by physiotherapists. These researchers used the psychological general wellbeing index questionnaire to assess HRQoL before and after the intervention and reported that participation in an exercise programme during pregnancy did not seem to influence HRQoL. However, our own study, which addressed a similar period of time, recorded very significant differences in HRQoL. This discrepancy may be due to the fact that the exercise programme in our case took place three days a week and was directed by specialised personnel. Contrasting data were also published by Petrov et al. [27], whose RCT of 92 healthy pregnant women, in which the EG performed moderate to vigorous exercise from weeks 14 to 25 of pregnancy, led them to conclude that the intervention had no significant impact on HRQoL.

Barakat et al. [28] conducted a study of 80 healthy pregnant women, in which the EG performed a programme of moderate exercise (35–45 min) for three days a week, from weeks 6–8 to 38–39 of pregnancy. These authors, like us, concluded that a programme of moderate physical activity during the first, second and third trimesters of pregnancy improves the mother’s perceived health status.

Vallim et al. [29] conducted a study very similar to ours, but with different results. Their trial was based on a study population of 66 women, among whom an EG (*n* = 31) received three classes per week of aerobic exercises in water, from weeks 20 to 36 of pregnancy. The women’ quality of life was measured with the WHOQoL-BREF questionnaire, and the results obtained led the authors to conclude that this exercise programme did not produce any differences in the HRQoL scores of the two groups, which were high in both the EG and in the CG. Although the latter study was similar to ours, it differed in the sample size. Thus, 66 healthy pregnant women began the trial, but only 43 completed it, producing a drop-out rate of 34.84%. Of the original 31 participants in the EG, 20 completed the exercise programme, for a drop-out rate of 35.48%. In our study, on the other hand, the dropout rate was 0% in the EG. Our findings indicate that although HRQoL worsened during pregnancy for all participants, the women in the EG experienced less deterioration in this respect than the more sedentary women.

Montoya et al. [30] conducted an RCT involving a programme of supervised exercise for healthy pregnant women, from weeks 16 to 32 of gestation, and concluded that HRQoL improved in the EG. These results therefore differed from ours in that these authors reported an improved HRQoL in the EG, while in our own case it worsened during pregnancy for both groups, although less so among the EG. The dropout rate reported by Montoya et al. (27.27%) also differed significantly from ours, which may have influenced the results obtained.

One of the most important strengths of this study is observed in the perinatal results, where we saw that the SWEP method has not influenced negatively the gestation time. As for beneficial effects, it is observed that the newborns have presented a significantly lower weight in the pregnant women who did physical exercise, with a difference of 200 g, but maintaining the weight of the newborns of both groups within the margin of normal weight of newborns. It has also been observed that women who performed the SWEP method had a higher percentage of optimal results in the state of the perineum, a higher percentage of intact pelvic floor after delivery and a lower percentage of tears and episiotomies.

Another of the strengths of this research is that in both groups few participants were lost to study during the process, thus enabling an optimum level of follow-up. On the other hand, difficulties were encountered in recruiting sufficient participants for the study, in part because the health services involved did not provide sufficient, appropriate information to respond to doubts raised by some women regarding physical exercise during pregnancy. Nevertheless, this limitation was overcome and did not influence the total sample size analysed.

The participants in this study were all women whose pregnancy did not present special risks, and therefore our results cannot be extrapolated to other cases in which such risks might exist.

Another possible limitation arises from the fact that the SF36v2 questionnaire is self-administered, which means there may be less internal consistency between this approach and that of questionnaires that are administered by interview, although an earlier study [31] observed no differences in this respect between the two methods.

## 5. Conclusions

Health care professionals should inform women of the HRQoL benefits of physical activity during pregnancy. The SF36v2 questionnaire measures eight dimensions of physical and mental health, but it should be taken into account that these conditions change naturally as the pregnancy advances [32].

Our study shows that women who perform physical exercise following the SWEP guidelines achieve better outcomes than sedentary women for various aspects of HRQoL. According to the SF36v2 scores obtained, the women who exercised suffered a less marked decrease in HRQoL, and in terms of the mental health component of this analysis, were not at risk of postpartum depression.

## Figures and Tables

**Table 1:** Baseline characteristics of the sample.

Characteristic	Exercise Group	Control Group	*p*-Value
	*n* = 65	*n* = 64	
Maternal age, years *	32.12 ± 4.43	30.58 ± 4.75	0.331
Min–max	21–43	22–43	
Previous children **	17(26.6%)	20(30.8%)	0.739
Marital status: married **	57(44.2%)	59(45.7%)	0.396
Height * (in centimetres; cm)	1.646 ± 0.06	1.651 ± 0.05	0.604
Weight first trimester * (in grams; g)	67.07 ± 12.23	67.89 ± 12.58	0.71
Weight third trimester * (in grams; g)	75.35 ± 12.13	79.05 ± 11.64	0.079
BMI first trimester ***	23.89[21.52–27.51]	24.01[21.78–26.58]	0.953
BMI third trimester *	27.76 ± 4.03	29.03 ± 4.45	0.092
Multiparous women **	20 (30.77)	17 (26.56)	0.739
**Domains of physical activity (according to intensity)**			
Daily work			0.88
Moderate **	57(44.2%)	56(43.4%)	
Vigorous **	4(3.1%)	3(2.3%)	
Moderate displacement **	41(31.8%)	44(34.1%)	0.497
Recreational activity			0.681
Moderate **	52(40.3%)	53(41.1%)	
Vigorous **	13(10.1%)	11(8.5%)	
**SF36v2 (week 12 of gestation); Mean ± SD**			
Physical Functioning *	84.15 ± 14.91	83.47 ± 12.59	0.769
Role-Physical *	65.86 ± 14.74	63.67 ± 13.77	0.384
Bodily Pain *	73.43 ± 16.72	75.36 ± 14.43	0.485
General Health *	77.06 ± 11.67	76.38 ± 15.31	0.775
Vitality *	64.32 ± 18.74	62.40 ± 14.96	0.52
Social Functioning *	88.65 ± 14.27	86.13 ±16.53	0.355
Role-Emotional *	47.43 ± 9.07	47.00 ± 12.18	0.82
Mental Health *	73.62 ± 15.01	73.98 ± 16.06	0.893
Physical Component Summary *	53.72 ± 4.56	53.46 ± 4.77	0.757
Mental Component summary *	44.86 ± 6.22	44.47 ± 7.13	0.747

* Mean ± SD, ** Frequency [%], *** Median [Q1–Q3].

**Table 2:** Descriptive data of the birth.

Characteristic	Exercise Group *n* = 65	Control Group *n* = 64	Total *n* = 129	*p*-Value
Days of Gestation * **Mean ± SD**	**280.09 ± 8.26**	**279.70 ± 8.92**	**279.90 ± 8.56**	***0.996***
Median [P25–P75]	281.00 [277.00–286.50]	281.00 [275.25–286.75]		
**Neonatal birthweight** * Mean ± SD (in grams; g)	3259.00 ± 564.40	3477.11 ± 414.51	3367.21 ± 505.79	0.011
Median[P25–P75] (in grams; g)	3250.00 [2955.00–3572.50]	3460.00 [3207.50–3770.00]		
**Perineum status**				
Whole perineum	26.15	3.12	<0.001	
Tear	38.15	42.19	0.469	
Episiotomy	35.4	45.31	0.146	
**Evolution of pregnancy** **Freq. (%)**				
Reasons for admission:				
Premature rupture of membranes **	22(33.8%)	18(28.1%)	0.776	
Childbirth in progress **	38(58.5%)	41(64.1%)		
Programmed **	5(7.7%)	5(7.8%)		
Start mode:				
Spontaneous start **	46 (70.8%)	39 (60.9%)	0.492	
Induced start **	14 (21.5%)	19 (29.7%)		
Others **	5 (7.7%)	6 (9.4%)		

* Mean ± SD, ** Frequency [%].

**Table 3:** Health-related quality of life during pregnancy.

	Control Group		Exercise Group		Group*	Time**	Time* Group***
SF36v2	12 Week	35 Week	12 Week	35 Week			
	Mean ± SD	Mean ± SD	Mean ± SD	Mean ± SD	*p*-Value	*p*-Value	*p*-Value
Physical Functioning	83.47 ± 12.59	67.81 ± 14.05	84.15 ± 14.91	77.15 ± 15.66	0.008	<0.001	0.012
Role Physical	63.67 ± 13.77	30.37 ± 11.12	65.86 ± 14.74	46.92 ± 12.16	<0.001	<0.001	<0.001
Bodily Pain	75.36 ± 14.43	55.02 ± 15.1	73.43 ±16.72	61.71 ± 18.04	0.316	<0.001	0.007
General Health	76.38 ± 15.31	70.3 ± 9.49	77.06 ± 11.67	76.14 ± 9.7	0.034	0.014	0.07
Vitality	62.4 ± 14.96	38.48 ± 13.69	64.32 ± 18.74	56.34 ± 15.49	<0.001	<0.001	<0.001
Social Functioning	86.13 ± 16.53	75.59 ± 13.07	88.65 ± 14.27	85.38 ± 15.08	0.003	<0.001	0.025
Role Emotional	47.00 ± 12.18	23.18 ± 8.96	47.43 ± 9.07	30.9 ± 7.47	0.002	<0.001	0.001
Mental Health	73.98 ± 16.06	70.7 ± 13.06	73.62 ± 15.01	73.77 ± 10.23	0.47	0.316	0.271
Physical Component Summary	53.46 ± 4.77	45.39 ± 4.21	53.72 ± 4.56	49.79 ± 4.59	0.001	<0.001	<0.001
Mental Component summary	44.47 ± 7.13	39.2 ± 4.16	44.86 ± 6.22	42.57 ± 5.16	0.016	<0.001	0.29

*Group: the effect of the group is taken into account., **Time: the effect of time is taken into account, ***time * group: the effect of time and group is taken into account simultaneously.
